# Modifiable factors related to life-space mobility in community-dwelling older adults: results from the Canadian Longitudinal Study on Aging

**DOI:** 10.1186/s12877-020-1431-5

**Published:** 2020-01-31

**Authors:** A. Kuspinar, CP Verschoor, MK Beauchamp, J. Dushoff, J. Ma, E. Amster, C. Bassim, V. Dal Bello-Haas, M. A. Gregory, JE Harris, L. Letts, S. E. Neil-Sztramko, J. Richardson, R. Valaitis, B. Vrkljan

**Affiliations:** 1 School of Rehabilitation Science, Faculty of Health Sciences, McMaster University, Hamilton, Ontario Canada; 2 Health Sciences North Research Institute, Sudbury, Ontario Canada; 3 Department of Health Research Methods, Evidence and Impact, Faculty of Health Sciences, McMaster University, Hamilton, Ontario Canada; 4 Department of Biology, Faculty of Science, McMaster University, Hamilton, Ontario Canada; 5 Department of History, Faculty of Humanities, McMaster University, Hamilton, Ontario Canada; 6 School of Nursing, Faculty of Health Sciences, McMaster University, Hamilton, Ontario Canada

**Keywords:** Older adults, Life-space mobility, Rehabilitation, Canadian longitudinal study on aging

## Abstract

**Background:**

The most common methods for measuring mobility in older adulthood include performance-based tests, such as the Timed-Up-and-Go and gait speed. While these measures have strong predictive validity for adverse outcomes, they are limited to assessing what older adults do in standardized settings, rather than what they do in their daily life. Life-space mobility, which is the ability to move within environments that expand from one’s home to the greater community, has been proposed as a more comprehensive measure of mobility. The aim of this study was to determine the association between modifiable factors and life-space mobility in older adults enrolled in the Canadian Longitudinal Study on Aging (CLSA).

**Methods:**

Life-space mobility was measured using the Life Space Index (LSI). Explanatory factors included physical, psychosocial and cognitive determinants, as well as pain, fatigue, driving status, nutrition, body mass index, smoking status, and vision. To estimate the association between the LSI and explanatory variables, univariate and multivariable ordinary least squares regression analyses were performed.

**Results:**

All adults 65 years and older (*n* = 12,646) were included in the analysis. Fifty percent were women and the mean age was 73.0 (SD5.7). The mean LSI score was 80.5, indicating that, on average, the sample was able to move outside of their neighborhood independently. All explanatory variables were significantly associated with the LSI except for balance and memory. The top 3 variables that explained the most variation in the LSI were driving, social support and walking speed.

**Conclusion:**

To our knowledge, this was the first study to examine the association between life-space mobility and a comprehensive set of modifiable factors that were selected based on a theoretical framework and existing research evidence. This study had two important messages. First, driving, social support and walking speed emerged as the most significant correlates of life-space mobility in older adults. Second, life-space mobility is multifactorial and interventions that are pragmatic in their design and testing are needed that consider the complexity involved. A multi-disciplinary approach to examining life-space mobility in older adults is needed to optimize opportunities for healthy aging and develop strategies that support mobility in older adulthood.

## Background

Declining mobility can be a significant challenge in older adulthood [1]. Changes in mobility at this life stage can range from underlying impairments in strength and joint movement to problems with accessing one’s community. In particular, physical mobility has received much attention in the geriatric and rehabilitation literature, as reflected in the most common measures used to capture this aspect of mobility. Such measures can include, but are not limited to the Timed Up and Go [2], gait speed [3], as well as self-reported problems with mobility, including difficulty walking [4]. While many of these measures have strong predictive validity for adverse health outcomes [5–7], they are focused on specific aspects of physical performance, and fail to recognize the interaction between individuals and their environment. Furthermore, such measures are limited to assessing what older adults do in standardized conditions, rather than what they are able to do in their everyday life [8]. Given these limitations, there has been a call for a more comprehensive conceptualization and corresponding means of measuring mobility [8, 9].

In a theoretical framework proposed by Webber, Porter and Menec (2010), mobility in older adulthood is presented as a complex and interrelated set of factors that come together to influence the ways in which seniors’ move within their home and community environments [9]. Using this framework, mobility-related impairments are recognized as limitations in one’s ability to access different life-spaces. The notion of life-space mobility, which is the ability for individuals to safely move within and across environments that expand outwards from their home to their wider community, has also been emphasized in aging research [9, 10]. Life-space mobility captures the range of physical environments (e.g., home, neighbourhood, town), or levels, in which individuals move during a specified time period [10]. Recent studies have demonstrated that reduced life-space mobility is an especially strong predictor of adverse outcomes in older adulthood, including falls, hospitalization, and even early death [11–13].

A considerable body of literature has identified a number of independent factors that are associated with deficits in physical function typically assessed using performance based measures [14–17]. However, there is limited research regarding the factors that are associated with life-space mobility. In order to inform rehabilitative and preventative strategies, it is important to understand the association between life-space mobility and factors that may be amenable and sensitive to change with intervention [17, 18]. For such an analysis, a large and representative population-based dataset can help identify factors that can impact life-space mobility. The Canadian Longitudinal Study on Aging (CLSA) [19] is one of the world’s largest population-based datasets that offers a unique opportunity to evaluate a comprehensive set of putative factors that can impact life-space mobility in the aging population. Therefore, the aim of this study was to determine the association between potentially modifiable risk factors and life-space mobility in older adulthood.

## Methods

### Cohort profile and participants

The current study involved a cross-sectional analysis of the CLSA baseline dataset (collected 2012–2015). The CLSA is a 20-year longitudinal study that includes over 50,000 community-dwelling adults aged 45 to 85 years at the time of the recruitment. Those with significant cognitive impairment (i.e. dementia), full-time members of the Canadian Armed Forces, individuals who resided in long-term care institutions, on Federal First Nations reserves or other First Nations settlements or in the Canadian territories, individuals who were not able to communicate in English or French were excluded at the time of recruitment [19]. The current analysis employed a sub-set of the CLSA Comprehensive Baseline Dataset (Version 4.0), which included 30,097 adults (aged 44–85) who completed in-person assessments at 1 of 11 CLSA data collection sites nationwide (i.e., Vancouver, Surrey, Victoria, Calgary, Winnipeg, Hamilton, Ottawa, Montreal, Sherbrooke, Halifax, St. John’s). This paper included all eligible participants aged 65 years and older (*n* = 12,646).

### Outcome variable

#### Life-space mobility

Life-space mobility was quantified using the Life Space Index (LSI) [10, 20], which is a self-reported measure of community mobility with strong psychometric properties [10, 11, 20, 21]. Specifically, the LSI measures the frequency and extent of movement within and from one’s home to the neighborhood and regions beyond. The LSI requires participants to report their level of mobility within and across different locations (i.e., 1 = rooms in the house outside of the bedroom, 2 = yard or immediate outdoor area, 3 = their neighbourhood, 4 = neighbourhoods outside their own, and 5 = outside of their city or town), their frequency of going there (i.e., 1 = < 1/week, 2 = 1–3 times/week, 3 = 4–6 times/week, and 4 = daily), and whether they needed assistance (i.e., 1 = personal assistance, 1.5 = assistive devices, 2 = no assistance) [10]. Scores within each level were multiplied, and summed to calculate the final LSI score (range 0–120), where a score of 120 characterizes the highest possible level of life-space (i.e. going out of town without assistance) [10]. A change of 5 to 10 points on the LSI is considered clinically meaningful [10, 22].

### Explanatory variables

Modifiable factors were selected using Webber’s conceptual framework of life-space mobility [9] and previous research evidence where factors that demonstrated an association with mobility were identified. Factors were categorized in accordance with Webber’s framework, including physical [17, 18, 23], psychosocial [24, 25] and cognitive determinants [26], alongside other factors that were highlighted in previous studies, such as pain [27], fatigue [28], driving status [29], nutrition [30, 31], body mass index [32], smoking status [33, 34] and vision [35].

### Physical

#### Walking speed

Walking speed was quantified using the Timed 4-Metre Walk Test [36]. Participants were positioned with their toes touching the starting line and instructed to walk at a typical pace until they passed the finish line located 4-m away. Participants observed an instructor-led demonstration and completed a single practice trial before data were collected.

#### Grip strength

Grip strength, a marker of overall muscle strength [37, 38], was quantified using standard handgrip dynamometry [39]. Participants used an electronic handgrip dynamometer to perform 3 maximal hand grip contractions with their dominant hand. Participants were evaluated while seated with their feet flat on the floor, dominant arm unsupported, with their elbow flexed at 90 degrees and the hand in a neutral position. The average of the maximal grip strength (kg) obtained across the 3 trials of their dominant arm were used for analysis.

#### Balance

Balance was measured using the Single Leg Stance test [40]. For this test, participants were timed while they performed a static one-legged stand, which required them to lift their given leg to the calf, bending their knee of the raised leg while placing their hands on their waist. The one-legged stand was performed first on the left leg and then the right leg (60 s maximum for each leg). The leg with the highest score was used for the analysis.

### Psychosocial

#### Depression

Depressive symptoms were evaluated using the Center for Epidemiological Studies Depression Scale Short Version 10 (CESD-10) [41]. The CESD-10 contains 10 questions regarding the presence of depressive feelings, loneliness, restless sleep, and hopefulness for the future over the preceding week. The maximum attainable score is 30, where higher scores indicate greater depressive symptomatology. A score of 10 or more on the CES-D is indicative of depressive symptoms [41].

#### Social support

The Medical Outcomes Study (MOS) Social Support Survey was used to determine social support [42]. This tool evaluates the degree by which interpersonal relationships serve meaningful functions across 4 subscales (i.e., emotional/information support, tangible support, affectionate support, positive social interaction), with answers ranging from 1 (none of the time) to 5 (all of the time). The MOS Social Support survey has a maximum score of 100, where higher scores indicate greater social support. Total MOS scores were categorized as a continuous variable for analyses.

### Cognition

Given that both language of administration and level of education attained can significantly impact resultant scores on cognitive testing [43] and that level of education has steadily increased over time, especially for women [44], t-scores were adjusted for language of administration (French or English) and level of education (less than high school, high school, some post-secondary or post-secondary) that were derived from the cognitive tests (i.e. Mental Alternation Test and Rey’s Auditory Verbal Learning Test) employed in the current study using a previously described approach [45].

#### Executive function

The Mental Alternation Test (MAT) [46, 47] was used to evaluate set-shifting abilities, as a measure of executive function. The MAT is an oral cognitive switching task that requires participants to alternate between the numbers 1 through 26 and the letters of the alphabet (i.e., 1-A-2-B-3-C, etc.), and has been shown to be sensitive and reliable in detecting global cognitive impairment [48]. The time to complete the MAT (seconds) was used for the current analyses with a maximal allowed score of 30 s.

#### Verbal learning and memory

Rey’s Auditory Verbal Learning Test was used to evaluate verbal learning and memory [49]. Participants were read a 15-item list of monosyllabic words and were required to accurately recite as many words from this list as possible immediately and 30 min later (i.e. delayed recall). The correct number of responses used in the delayed recall (REYII) was used for this analysis [50].

### Pain

#### Pain

Pain was quantified using a single item from the CLSA “Are you usually free of pain or discomfort?” which has a binary response option of “yes” or “no.”

### Fatigue

#### Fatigue

Fatigue was operationalized using a single item “About how often during the past 30 days did you feel tired out for no good reason —would you say all of the time, most of the time, some of the time, a little of the time, or none of the time?” A binary variable was derived from the responses, where all of the time and most of the time were coded as “yes” and the rest were coded as “no”.

### Vision

#### Self-reported vision

Self-reported vision was measured using a single item “Is your eyesight, using glasses or corrective lens if you use them… poor, fair, good, very good, or excellent.” Responses were coded to create a binary variable, poor/fair vs. good/very good/excellent.

### Driving

#### Driving status

Driving status was operationalized using the CLSA Transportation, Mobility, and Migration module. This module was adapted from the Baseline Survey of Seniors (Older and Wiser Driver Questionnaire) [51]. The questions in this module capture various aspects of transportation mobility, including driver’s license status alongside their perceived behind-the-wheel behaviour in certain driving situations, such as not driving on highways, during rush hour or at night (i.e., situational avoidance). Questions in the CLSA that asked participants about their situational avoidance when driving have demonstrated good test-retest reliability in studies with multiple samples [52, 53]. For the purpose of this analysis, each participant was categorized into 3 groups: i) not licensed, ii) licensed with ≥1 reported area of situational avoidance (e.g., avoid driving on highways, rush hour, left hand turns, at night), or iii) licensed but with no situational avoidance behaviours.

### Nutrition

#### Nutritional risk

Nutritional risk was measured using the Abbreviated Seniors in the Community Risk Evaluation for Eating and Nutrition (SCREEN II) questionnaire [54]. This tool contains 8 questions related to typical daily eating habits (i.e., weight gain or loss, skipped meals, difficulty eating, etc.). Scores for each item were summed to create an overall SCREEN II score (max score 48) that was used for this analysis.

### Body mass index

#### Body mass index

Body mass index (BMI) was calculated using an individual’s weight in kilograms divided by the square of an individual’s height in metres acquired by trained personnel during the in-person visit. Underweight (< 18.5), normal weight (18.5 to 24.9), overweight (25.0 to 29.9), obese class I (30.0–34.9), obese class II (35.0–39.9), and obese class III (> 40.0) were defined according to the World Health Organization’s BMI nutritional status categories.

### Smoking

#### Smoking status

Smoking status was divided into 3 categories: non-smokers (people who had never smoked a whole cigarette), smokers (people who are currently daily or occasional smokers) and former (people who were formerly daily or occasional smokers). Past population-based studies have demonstrated that smoking is associated with mobility loss in older adults [34, 55].

### Covariates

Personal factors known to influence mobility in aging, but that are not directly modifiable by intervention or rehabilitation, were selected as covariates. These variables were: age [56], sex [57], education [58], income [59], number of chronic conditions [60], marital/partner status [61], and residential location (rural vs. urban) [62]. These factors were included in our full regression model but their relationships with the LSI are not reported in the paper.

### Statistical analysis

All analyses were performed in R v3.6.0. Descriptive statistics, including mean and standard deviation for continuous variables and count/frequency for categorical variables, were used to describe the distribution of all variables included in this study. To estimate the association between LSI and the explanatory variables of interest, both univariate and multivariable ordinary least squares regression analyses were performed. To facilitate comparability between variables, continuous explanatory variables were standardized prior to analysis, and missing data was removed. For our multivariable analysis, a single full model including all explanatory variables and covariates was employed; our decision to include all variables in the multivariable model was made a priori, and was contingent on the model not violating any assumptions. Multicollinearity between independent variables was found to be minimal (variance inflation factor < 3), no high leverage (i.e. according to Cook’s distance) data points were identified and residuals were found to be approximately normal. A minor departure from homoscedasticity was observed, hence, robust standard errors were calculated using an HC3 estimator [63]. To evaluate which explanatory variables contributed the greatest to variation in LSI in our full model, we used the R package ‘relaimpo’ [64]. Relative importance of each variable was calculated using the “variables added last” approach and reported as the percentage of the total variation explained.

## Results

Tables 1 and 2 outline the characteristics of the sample (*n* = 12,646). Almost 50% of participants were women and the mean age was 73.0 (Standard Deviation 5.7) of which 71.4% had a post-secondary degree and the majority (91.7%) lived in an urban environment. The mean LSI score was 80.5 (out of 120), indicating that, on average, the sample was able to move outside of their neighborhood independently [65].

**Table 1:** Sample characteristics (*n* = 12,646) described using categorical variables

Characteristic	N (%)
Female	6306 (49.9)
Male	6340 (50.1)
Number of chronic conditions	
0	902 (7.1)
1	1899 (15)
2	2199 (17.4)
3	2144 (17)
4 or more	5457 (43.2)
NA	45 (0.4)
Income	
< 20 K	811 (6.4)
20-50 K	3915 (31)
50-100 K	4534 (35.9)
100-150 K	1505 (11.9)
150 K+	753 (6)
NA	1128 (8.9)
Education	
Less than secondary school	1153 (9.1)
Secondary school	1390 [11]
Some post-secondary	1039 (8.2)
Post-secondary degree	9026 (71.4)
NA	38 (0.3)
Rural	858 (6.8)
Urban	11,601 (91.7)
NA	187 (1.5)
Marital status/Partner	
Single	739 (5.8)
Married	7875 (62.3)
Widowed	2313 (18.3)
Divorced	1483 (11.7)
Separated	235 (1.9)
NA	1 (0)
Smoker	
Non-smoker	6208 (49.1)
Smoker	555 (4.4)
Former	5786 (45.8)
NA	97 (0.8)
BMI	
Normal	3534 (27.9)
Under	106 (0.8)
Over	5397 (42.7)
Obese I	2499 (19.8)
Obese II	744 (5.9)
Obese III	305 (2.4)
NA	61 (0.5)
Depression	
No	10,657 (84.3)
Yes	1874 (14.8)
NA	115 (0.9)
Fatigue	
No	11,174 (88.4)
Yes	821 (6.5)
NA	651 (5.1)
Pain	
No	7275 (57.5)
Yes	4764 (37.7)
NA	607 (4.8)
Vision	
Good/Very Good/Excellent	11,492 (90.9)
Poor/Fair	1142 (9)
NA	12 (0.1)
Driving	
Not Driving	988 (7.8)
Driving without situational avoidance	2131 (16.9)
Driving with situational avoidance	8932 (70.6)
NA	595 (4.7)

NA: Missing

**Table 2:** Sample characteristics (n = 12,646) described using continuous variables

Characteristic	% Missing	Mean	Standard Deviation	Minimum	Maximum
Age	0.00	73.1	5.69	65	86
Life-Space Mobility (LSI)	0.00	80.5	18.37	0	120
Executive Function (MAT)	0.06	24.2	8.62	0	51
Memory (REY II)	0.05	3.3	1.99	0	13
Nutrition (SCREEN II)	0.08	38.9	6.00	5	48
Balance (Single Leg Stance in sec)	0.09	21.5	21.80	0	60
Grip Strength (kg)	0.10	30.0	10.3	0.16	73.68
Walking speed (m/s)	0.02	0.9	0.20	0.20	2.33
Social Support (MOS Social Support Survey)	0.03	80.0	17.8	0	100

*MAT and REYII are not adjusted for language and level of education

With regard to the LSI, 77.8% of participants reported being able to get to places beyond their town (Table 3). Of these participants, 95.0% were completely independent in their mobility, 3.5% used a mobility aid (e.g. walker), and 1.5% needed assistance from another person. Twenty-two percent of the sample reported being able to access their neighborhood but stayed within the confines of their town. Of these individuals, 93.7% did not need any assistance, 5.0% used a mobility aid and 1.4% needed the assistance of another person.

**Table 3:** Highest life-space reached and level of assistance required

	Within the home	Areas outside the home (e.g. porch, deck)	Within the neighborhood	Outside neighborhood within the same town	Outside the town
Highest life-space level reported	0%	0%	0.5%	21.7%	77.8%
LEVEL OF ASSISTANCE					
None	97.6%	96.2%	94.3%	93.7%	95.0%
Equipment only	2.3%	3.6%	5.0%	5.0%	3.5%
Personal assistance	0.1%	0.2%	0.7%	1.4%	1.5%

Table 4 outlines the results of the univariate and multivariable regression analysis. In the univariate analysis, all explanatory variables were significantly associated (*p* < 0.0001) with the LSI score. Higher rates of walking speed, balance, grip strength, nutrition, social support, executive function and memory were associated with a higher score on the LSI. For example, an increase in walking speed and grip strength by 1 standard deviation, resulted in an increase of 4.98 (95% CI 4.67, 5.28) and 4.71 (95%CI 4.39, 5.03) points, respectively on the LSI. Similarly, better nutrition and social support was associated with a 3.22 (95%CI 2.95, 3.49) and 3.47 (95%CI 3.16, 3.79) point increase on the LSI, respectively. Driving, compared to not driving, was associated with an increased score on the LSI of 23 (95%CI 21.24, 23.85) points. On the other hand, a BMI of less than 18.5 (underweight), and a BMI of greater than 30.0 (obesity), smoking, pain, fatigue, poor vision and depressive symptoms were associated with lower LSI scores. For example, the presence of pain and fatigue, decreased LSI scores by approximately 5 and 10 points, respectively.

**Table 4:** Univariate and multivariable regression analysis assessing the association between each modifiable factor and the Life Space Index

Variable	Univariate Coefficient (95% CI)	R^2^	Multivariable Coefficient (95% CI)
Walking			
• Walking speed^§^	4.98 (4.67, 5.28)***	0.076	1.07 (0.65, 1.49) ***
Balance			
• Single Leg Stance^§^	3.05 (2.74, 3.36) ***	0.031	0.35 (−0.03, 0.74)
Grip			
• Grip strength^§^	4.71 (4.39, 5.03) ***	0.068	1.08 (0.52, 1.64) ***
Body Mass Index			
• Normal	Ref	0.016	Ref
• Under	−7.52 (−11.01, − 4.03) ***		− 5.2 (− 10.17, − 0.23)*
• Over	1.25 (0.48, 2.02) **		0.96 (0.13, 1.78)*
• Obese I	−1.21 (−2.14, −0.29) *		0.62 (− 0.45, 1.68)
• Obese II	−4.15 (− 5.59, − 2.72) ***		− 0.37 (− 2.23, 1.49)
• Obese III	− 11.39 (− 13.51, − 9.28) ***		−2.01 (− 5.12, 1.1)
Smoking			
• Non-smoker	Ref	0.006	Ref
• Smoker	−6.9 (−8.49, −5.3) ***		−3.37 (− 5.42, − 1.32)**
• Former	− 0.19 (− 0.84, 0.47) ***		− 0.26 (− 0.98, 0.46)
Nutritional Risk			
• Nutritional Risk^§^	3.22 (2.95, 3.49) ***	0.032	0.53 (0.12, 0.95)*
Pain			
• No	Ref	0.019	Ref
• Yes	−5.07 (−5.73, −4.41) ***		−1.05 (− 1.81, −0.29)**
Fatigue			
• No	Ref	0.018	Ref
• Yes	−9.62 (−10.9, −8.34) ***		−1.99 (−3.68, −0.31)*
Vision			
• Good/Very Good/Excellent	Ref	0.013	Ref
• Poor/Fair	−7.19 (−8.31, −6.08) ***		−2.08 (−3.53, −0.63)**
Driving			
• Not Driving	Ref	0.091	Ref
• Driving without situational avoidance	22.55 (21.24, 23.85) ***		9.67 (7.86, 11.47)***
• Driving with situational avoidance	18.20 (17.06, 19.34) ***		8.02 (6.38, 9.66)***
Social Support			
• MOS Social Support Survey^§^	3.47 (3.16, 3.79) ***	0.036	1.37 (0.95, 1.79)***
Depressive Symptoms			
• No	Ref	0.026	Ref
• Yes	−8.24 (−9.12, −7.35) ***		−1.65 (−2.79, −0.51)**
Executive Function			
• Mental Alternation Test^§^	2.36 (2.04, 2.69) ***	0.017	0.41 (0.03, 0.8) *
Memory			
• REYII^§^	0.47 (0.15, 0.80) **	0.001	−0.23 (−0.62, 0.15)
TOTAL R^2^			**0.135**

Multivariable model is adjusted for age, sex, education, income, residing in urban/rural, lives with partner, and number of chronic conditions

Ref: reference category

^§^Higher scores indicate better performance on the measure

*** < 0.001, ** < 0.01, * < 0.05

**Fig. 1:**
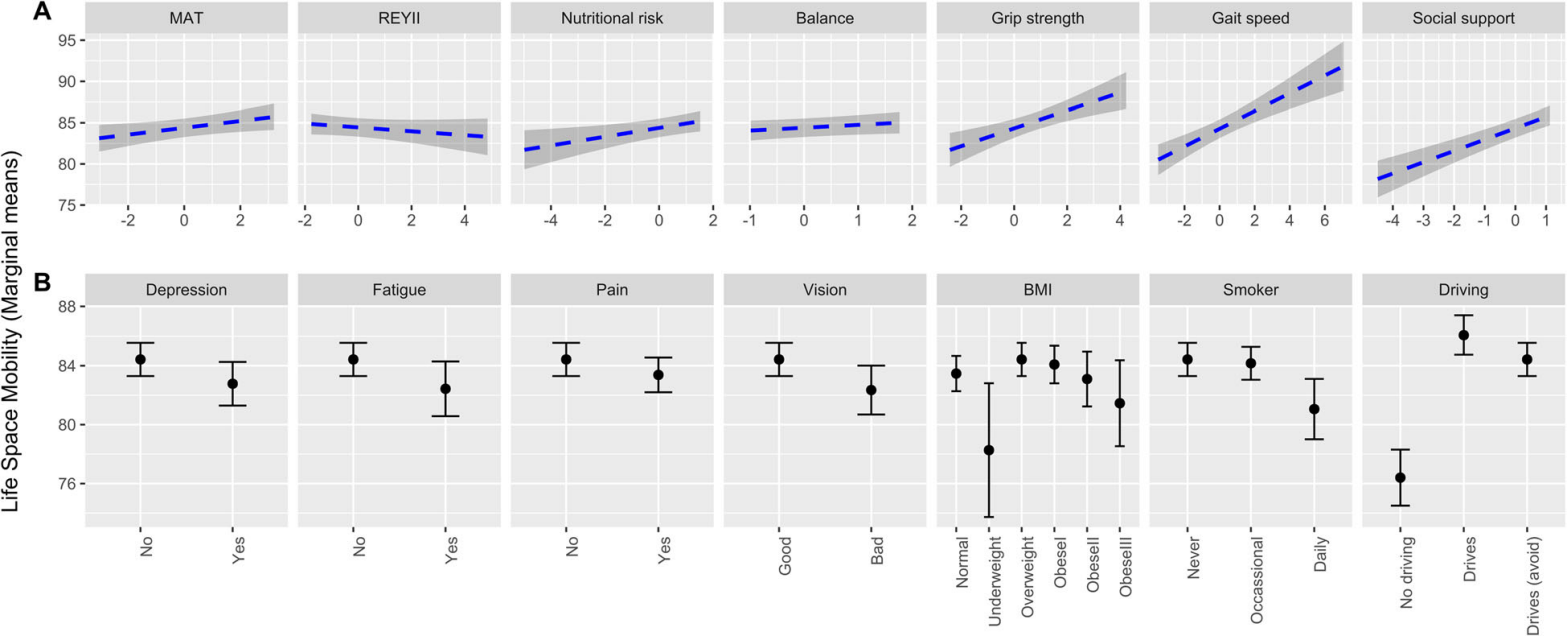
Conditional effects of our explanatory variables of interest on life-space mobility. Conditional effects were determined for **a**) standardized continuous and ) categorical explanatory variables using a fully adjusted multivariable model (including all explanatory variables and covariates). For A, the units for the x-axis are standard deviations from the mean, and for B, the reference category for each individual plot is listed first. The significance of the regression slope (**a**) or difference from the reference category (**b**) can be found in Table 4

In the multivariable regression model (Table 4; Fig. 1) all explanatory variables were significantly associated with the LSI except for balance and memory. While current smokers exhibited a mean LSI that was at least 3 points lower than non-smokers, the scores of former smokers were not significantly different from non-smokers. Being underweight was associated with a 5-point reduction in LSI, as compared to normal weight. Having a license with or without enacting situational avoidance behaviours was associated with an LSI increase of greater than 8 points. All other variables (walking speed, grip strength, nutritional risk, pain, fatigue, vision, social support, depressive symptoms and executive function) were associated with an increased or reduced LSI of approximately 2 points or less. Collectively, the explanatory variables explained 13.5% of the variance in the LSI.

**Fig. 2:**
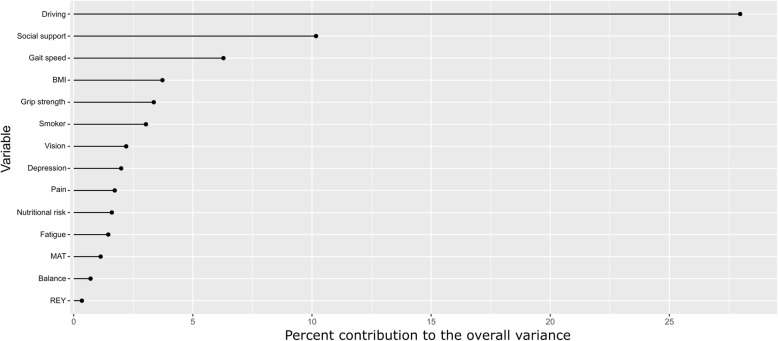
Each explanatory variable ordered from most important to least important

Figure 2 is a graphical depiction of the explanatory variables that contributed the greatest to the variation in LSI in the multivariable model. The top 3 variables that explained the most variation in the LSI were driving, social support and walking speed followed by BMI, grip strength, smoking, vision, depressive symptoms, pain, nutritional risk, fatigue, executive function, balance and memory.

## Discussion

To our knowledge, this was the first study to examine the association between life-space mobility and a comprehensive set of modifiable factors that were carefully selected based on a theoretical framework and existing research evidence. When all of the modifiable factors were compared in one regression model, those that can significantly influence life-space mobility emerged. Specifically, driving, social support and walking speed were identified as the most important contributors to life-space mobility in older adulthood and explained the most amount of variance in terms of LSI scores.

For older adults in westernized countries, including Canada, driving has been identified as the most popular and preferred means of community mobility [66]. Having a driver’s license alongside access to a private automobile in older adulthood has been linked to higher rates of social participation [67]. Conversely, driving cessation in later life, whether voluntary or otherwise, can lead to adverse outcomes, including reduced out-of-home activity levels [68], decreased health status [69], higher rates of depression [70], institutionalization (e.g., long term care admissions) [71], and even death [72]. Although the LSI, as a measure, is less focused on the mode by which individuals access their immediate and surrounding environments, findings from the current study demonstrate the strength of the association between being a driver, even if one self-restricts their driving patterns, and life-space in older adulthood. Our findings also align with emerging evidence from a longitudinal study of 2792 community-dwelling older adults aged 65–94, with a similar mean age at study outset (i.e., 73.6 SD5.9) as the current investigation [73]. Non-drivers in their study were found to have a smaller life-space across time, but that differences in residential context might have a role, meaning non-drivers in low population density areas had significantly smaller life-spaces than those living in more highly populated locations. While this study explicates the impact that factors, such as place of residence, can have on life-space alongside driving status, these factors may not be easily amenable to change given that established social support networks are often linked to where people live. Nonetheless, it does demonstrate as do findings from the current study, the importance of having feasible transportation alternatives in place to support community mobility and participation.

Previous studies have also demonstrated an association between social support and out-of-home mobility in older adults. Using an ethnographic study design, Gardner (2014) explored older adults’ perspectives regarding the extent to which social factors predict community mobility in the aging population [74]. Gardner demonstrated how social interaction can positively influence community mobility by encouraging older adults to get outside their home despite the health challenges they may be experiencing. Furthermore, Mclaughlin and colleagues demonstrated in a mixed-methods study that seniors with larger social networks were more likely to report having higher levels of mobility [75]. These studies, along with results from the current analysis, highlight the importance of having social support in later life.

From the analysis, another important factor that can impact life-space mobility was walking speed, which was measured by asking an individual to walk a predefined distance at their self-selected pace. Walking speed has been shown to be a predictor of a range of outcomes including falls [76], institutionalization [77], hospitalization [78] and death [6]. Some researchers have referred to walking speed as the ‘sixth vital sign’ due to the growing body of evidence suggesting it is indicative of overall health [79]. While our findings are similar to that of Peel and colleagues who demonstrated a relationship between walking speed and the LSI in a sample of 1000 community-dwelling older adults [20], our study is based on a considerably larger, random and population-based sample.

Although driving, social support and walking speed had the strongest effect on life-space mobility, other factors, such as grip strength, vision, depression, pain, nutritional risk and smoking status, were also associated with life-space mobility. These results demonstrate that life-space can be influenced by a multitude of factors, which has important implications for clinical practice. Healthcare providers should consider these factors when assessing and treating those with mobility limitations. For example, recognising pain as a factor that can negatively affect life-space mobility, determining the best interventions, particularly those that are non-pharmacological in nature, such as exercise programs which can be tailored to the needs of the older person, should be considered.

Interestingly, cognitive function, executive function and memory did not emerge as strong predictors of life-space mobility in this study. The weak association could be due to the fact that the CLSA sample has not yet exhibited impairments in cognitive functioning to the point that life-space mobility is affected. However, this association may be different for other subdomains of cognitive function such as processing speed and attention.

A strength of this study was its examination of the association between a comprehensive set of modifiable factors with life-space mobility in older adults that were determined in accordance with the best research evidence, including a theoretical framework focused on aging and mobility. To our knowledge, no study to date has been as comprehensive in scope when examining factors amenable to change when it comes to the life-space mobility of community-dwelling older adults. Furthermore, the large and randomized sample of the population-based CLSA dataset provides a unique opportunity to closely examine the relationship between each explanatory factor and life-space mobility within the same model. However, a potential limitation of our analysis was the use of cross-sectional data. At the time this study was undertaken only baseline data was available from the CLSA. Hence, the current study sets the stage for future longitudinal analysis where the relationship between these factors and life-space mobility can be tracked. Furthermore, the multivariable model was able to explain only 13.5% of the variance in the LSI, leaving 86.5% of the variance unexplained. Hence, there may be other factors that can affect life-space mobility, including community design (e.g. safety and security, green space, design of sidewalks and streets), accessibility of activities beyond the home, anxiety and motivation. Life-space mobility may also be influenced by the presence of sarcopenia, as suggested by the work of Curcio and colleagues, and is related to falls and physical inactivity in non-institutionalized older adults [80, 81]. While we were able to partially account for sarcopenia in our models though the incorporation of grip strength and gait speed, additional factors, such as lean mass, may have improved the proportion of variance in LSI explained. Clearly, future research should examine the extent to which such factors like sarcopenia are associated with life-space mobility in community-dwelling older adults.

## Conclusion

In conclusion, this study has two important messages. First, driving, social support and walking speed emerged as the most significant correlates of life-space mobility in Canadian older adults. However, these associations were derived using cross-sectional data, and as such, it is unclear whether life-space mobility could improve by addressing these factors through targeted interventions. Second, life-space mobility is multifactorial and interventions that are pragmatic in their design and testing are needed that consider the complexity involved. A multi-disciplinary approach to examining life-space mobility in older adults is needed to optimize opportunities for healthy aging and develop strategies that support mobility in older adulthood.

## Data Availability

The data used for this study cannot be made available by the authors, as Canadian Longitudinal Study on Aging (CLSA) data are released to researchers only with approval of the CLSA Data Access Committee for a specific project. Further information on the data access can be found at https://www.clsa-elcv.ca/data-access.

## Abbreviations

BMI: Body mass index
CESD-10: Center for Epidemiological Studies Depression Scale Short Version 10
CI: Confidence Interval
CLSA: Canadian Longitudinal Study on Aging
LSI: Life Space Index
MAT: Mental Alternation Test
MOS: Medical Outcomes Study
REYII: Rey’s Auditory Verbal Learning Test delayed recall
SCREEN II: Abbreviated Seniors in the Community Risk Evaluation for Eating and Nutrition
SD: Standard Deviation
